# Bleeding of Perivascular Spaces in Midbrain of a Young Patient With Head Trauma

**DOI:** 10.7759/cureus.12884

**Published:** 2021-01-24

**Authors:** Federica Galli, Marco Pandolfi, Alessandro Liguori, Martina Gurgitano, Maurizio Sberna

**Affiliations:** 1 Radiology, Istituto Clinico Città Studi di Milano, Milan, ITA; 2 Radiology, Fondazione IRCCS ASST Ca’ Granda Ospedale Maggiore Policlinico, Milan, ITA; 3 Neuroradiology, ASST Grande Ospedale Metropolitano Niguarda, Milan, ITA

**Keywords:** perivascular space, head trauma, subarachnoid space, subarachnoid hemorrhage

## Abstract

Perivascular spaces (PVSs) surrounding the walls of arteries, arterioles, and venules are a common finding in brain imaging. Even if they do not directly communicate with subarachnoid spaces, there are some cases in which subarachnoid hemorrhage (SAH) and intracerebral hematomas extend to the PVSs by leakage of the leptomeninges. In this report, we present a case of enlargement and bleeding of PVSs in the midbrain of a young woman with head trauma, without evident SAH or intracerebral hematomas.

## Introduction

According to Hutchings and Weller [1] and Oztürk and Aydingöz [2], perivascular spaces (PVSs) surrounding the walls of arteries, arterioles, and venules do not directly communicate with subarachnoid spaces. There are a few reports exhibiting subarachnoid hemorrhage (SAH) and intracerebral hematomas extending to the PVSs by leakage of the leptomeninges that surround arteries [3-4]. A review discussed the role of mild traumatic brain injury as a cause of Virchow-Robin spaces (VRSs) pathological enlargement [5]. To our knowledge, this is the first report of enlargement and bleeding of PVSs in the midbrain of a young woman with head trauma, without SAH or intracerebral hematomas.

## Case presentation

**Figure 1:**
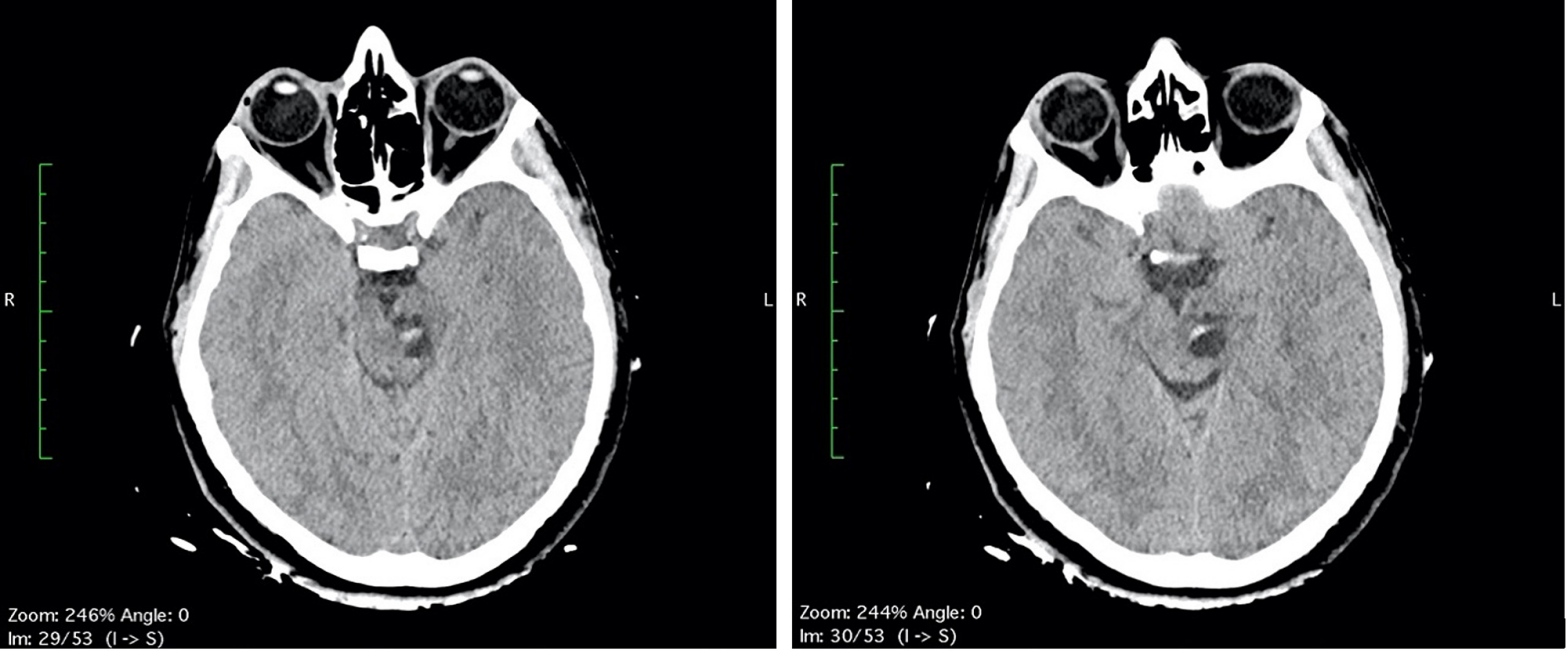
(A-B) Head CT scan: a multiloculated cyst in the left midbrain with eccentric hyperdensity, hematic-like.

A 42-year-old woman, without previous disease, was admitted in the Emergency Room for a polytrauma during an accident car. Clinically she was conscious; she had a head trauma with hairy scalp; she complained headache and pain of the left foot; on the accident set, opiates were administered. Pressure of oxygen (PaO2) was 98%, cardiac frequency was 65 bpm, Glasgow Coma Scale was 15, and the neurological examination was negative. Laboratory tests revealed hemoglobin: 10.7 g/dL (normal range 12-16 g/dL), white blood cell: 11.71 x 109/L (normal range 4-10 x 109/L), coagulation tests in normal range, and normal platelet count. A total body CT was performed; it demonstrated, beyond the hairy scalp with large soft tissue loss in frontoparietal zone, a multiloculated cyst in the left midbrain, with eccentric hyper-density, hematic-like (Figure 1). An X-ray of the left foot showed a fracture of the first toe. In absence of neurological deficit, the patient immediately underwent surgery for the reconstruction of the scalp. 

**Figure 2:**
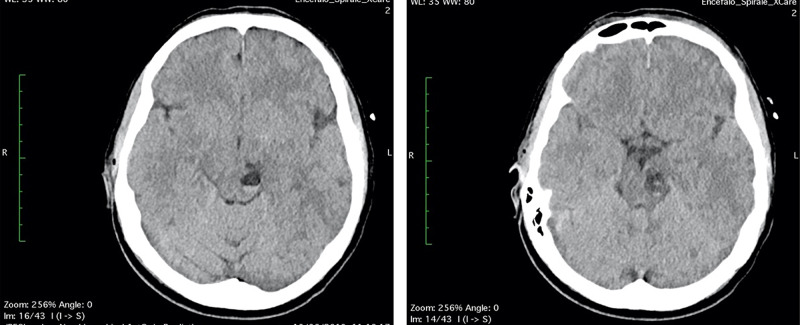
(A-B) Head CT scan “the day after”: the multiloculated cyst in the left midbrain showed fluid-fluid level, with enlargement of left cerebral peduncle and a slight mass effect, referring to enlargement and bleeding of PVSs. PVSs, perivascular spaces

**Figure 3:**
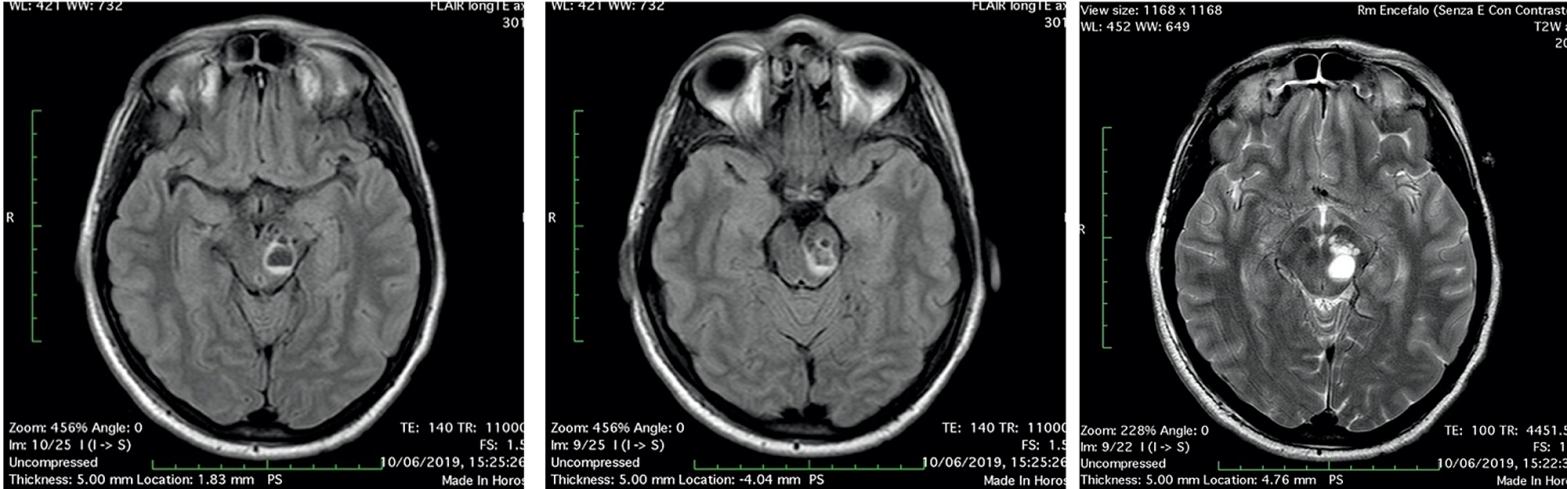
Head MRI scan “the day after”: the multiloculated cyst in the left midbrain showed fluid-fluid level, with enlargement of left cerebral peduncle and a slight mass effect, referring to enlargement and bleeding of PVSs (3A-3B: FLAIR axial image; 3C: T2 axial image). PVSs, perivascular spaces

The first day after surgery, the patient was transfunded for hemoglobin 6.8 g/dL (normal range 12-16 g/dL), referring to scalp surgery. She complained headache, vertigo, nausea and diplopia, reflecting an involvement of the brainstem and the oculomotor nerves. A second CT and brain MRI with IV contrast were performed: CT scan showed a fluid-fluid level, with enlargement of left cerebral peduncle and a slight mass effect (Figure 2). MRI scan confirmed the same findings of fluid-fluid level, with enlargement of left cerebral peduncle and a slight mass effect; no contrast enhancement was observed: these findings were referred to enlargement of VRSs with post-traumatic bleeding. Neither SAH nor intracerebral hematomas were revealed (Figure 3). No neurosurgery was indicated but only clinical and imaging follow up.

**Figure 4:**
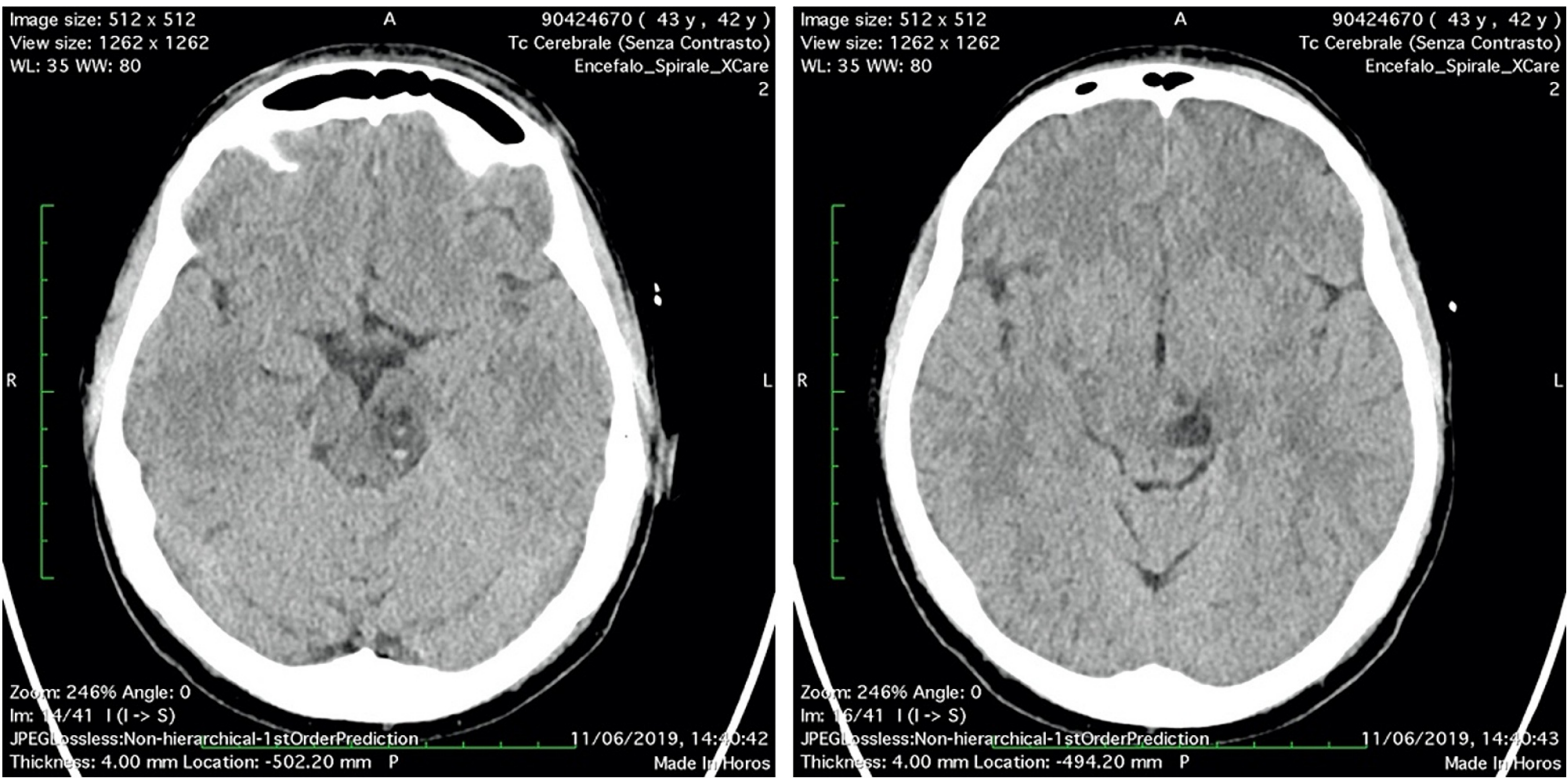
(A-B) Head CT scan “two days after”: no significant changes of the bleeding PVSs in the left midbrain. PVSs, perivascular spaces

Two days after the surgery, diplopia and nausea were disappeared, vertigo persisted. The patient stayed six days more in hospital to follow the reconstruction of the scalp and to recover. Unfortunately, the patient had a reject of transplanted skin with necrosis and had to schedule a new surgery in a month later. Before discharge, a CT scan was performed in order to assess the midbrain lesion (Figure 4).

**Figure 5:**
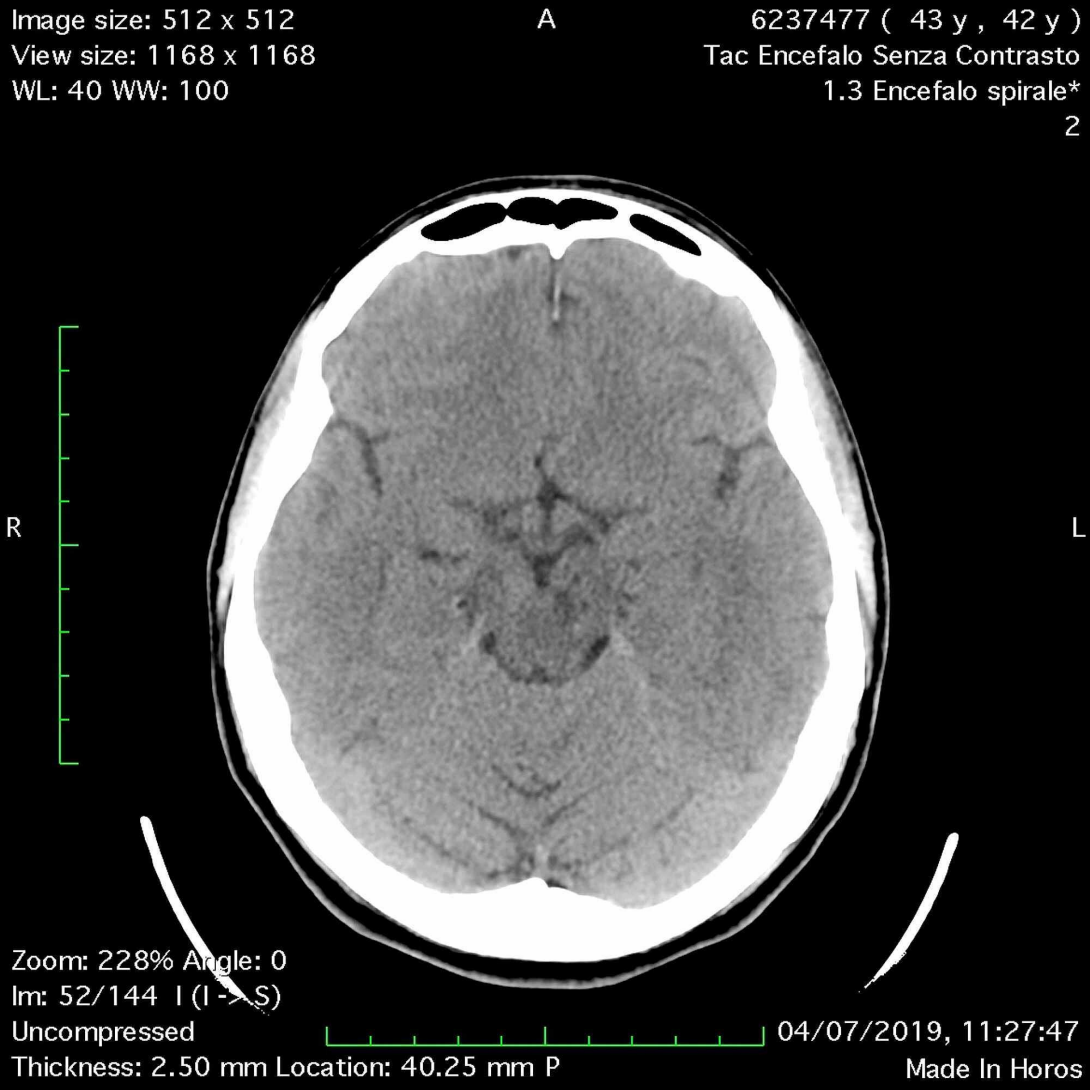
Head CT scan “after obe month”: the cystic lesion was reduced, with a punctiform residual hyperdensity.

**Figure 6:**
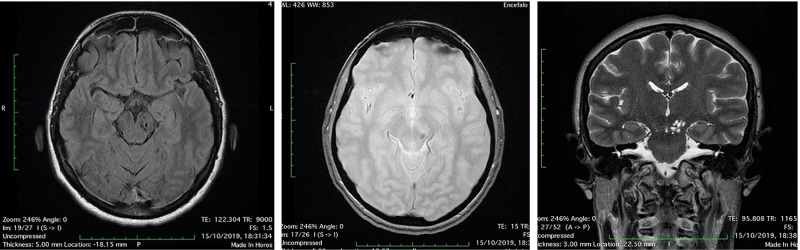
Head MRI scan “after four months”: the cystic lesion was reduced, with punctiform hemosiderin sign (A: FLAIR axial image; B: T2 gradient echo axial image; C: T2 coronal image).

The patient was discharged and she continued the treatment for the scalp lesion and gradually recovered from vertigo. She repeated CT and MRI exams to follow the midbrain lesion up: a CT scan after one month (Figure 5) and a MRI scan after four months (Figure 6). A significant volume reduction of the midbrain lesion was observed.

## Discussion

The PVSs were described for the first time by Rudolf Vircow and Charles Philippe Robin, in 1851 and 1859 respectively [6-7]. So far, they have been considered to be the continuation of subarachnoid space; however, the result of electronic microscopy and tracer study showed that the subarachnoid space did not communicate directly with the cerebral cortical PVSs [1]. Oztürk et al. confirmed that, comparing MR signal intensity of PVSs and the intensity of subarachnoid spaces [2]; they found that the mean signal intensity value of PVSs was statistically significantly lower than the intensity of the cerebrospinal fluid both in the subarachnoid space and in ventricles. In literature, there are a few reports exhibiting SAH and intracerebral hematomas extending to the PVSs by leakage of the leptomeninges that surround arteries [3-4]. In this report, we show a case in which the revealed blood is only inside the PVSs; indeed, we have not found any evident sign of SAH or intracerebral hematoma, which could have extended to the PVSs. To our knowledge, this is the first report in which the blood was apparently confined in the subpial space and not in the subarachnoid space. We can only suppose that it could be a bleeding in the subpial space, as it is possible in the subarachnoid space, and that it could be a distinct entity; alternatively, the patient could have had a SAH not detectable with CT and/or MRI imaging, which has extended to PVSs.

A recent review discussed the role of mild traumatic brain injury as a cause of VRSs pathological enlargement [5]. Even in our case, we had an enlargement of VRS in the midbrain after a head trauma. The resulting mass effect could explain the neurological symptoms that our patient complained. We can also notice that these findings of enlarged spaces reduce after one month from the trauma: even if this result is in contrast with a previous investigation reported in literature [8] that showed permanent PVSs dilations after mild traumatic brain injuries, maybe it can be explained by the concomitant bleeding as an additional cause of enlargement. 

In the end, we can speculate about possible differential diagnosis of our case. In 2007 a review described PVSs at MRI [9], exhibiting three typical locations of PVSs: the I type along the lenticulostriate arteries entering the basal ganglia through the anterior perforated substance, the II type along the paths of the perforating medullary arteries as they enter the cortical grey matter over the high convexities and extend into the white matter, the III type in the midbrain. In our case we have PVSs of III type, in the midbrain in the left cerebral peduncle, in one of the typical location. Nevertheless, we know that big dilatation of the PVSs may cause mass effect and assume uncommon configurations that could be misinterpreted as a cystic brain tumor [10-11]. However, cystic brain tumors often have solid components, may enhance with contrast material, mostly show surrounding edema, and have contents that usually are not equal to cerebrospinal fluid; these findings are not present in our case. We also could soon exclude other differential diagnosis, reported in literature [12], as cystic infection or chronic lymphocytic inflammation, according to our patient’s anamnesis.

## Conclusions

This case shows enlargement of PVSs in the midbrain of a young woman with head trauma, with a peculiar bleeding only inside these PVSs. In literature, there are some cases reporting bleeding of PVSs, but they were associated with the presence of SAH and intracerebral hematomas, extending to the PVSs by leakage of the leptomeninges. To our knowledge, this is the first report of enlargement and bleeding of PVSs in the midbrain after brain injury, without evident SAH or intracerebral hematomas.
